# Editorial: Advances in Plasma Cells in Health and Disease

**DOI:** 10.3389/fimmu.2020.606737

**Published:** 2020-10-21

**Authors:** F. Eun-Hyung Lee, Jens Wrammert, Simone Cenci

**Affiliations:** ^1^Division of Pulmonary, Allergy, Critical Care, and Sleep Medicine, Department of Medicine, Emory University, Atlanta, GA, United States; ^2^Lowance Center for Human Immunology, Emory University, Atlanta, GA, United States; ^3^Emory Vaccine Center, Emory University School of Medicine, Atlanta, GA, United States; ^4^Division of Infectious Disease, Department of Pediatrics, Emory University School of Medicine, Atlanta, GA, United States; ^5^Unit of Age Related Diseases, Division of Genetics and Cell Biology, Ospedale San Raffaele, Milano, Italy; ^6^University Vita-Salute San Raffaele, Milano, Italy

**Keywords:** niche, bone marrow, allergy, serological memory, plasma cell, multiple myeloma, immunoglobulin, antibody

The plasma cell (PC) is a relatively fledging cell type in biomedical science because its functional recognition dates back only to the mid 20^th^ century (1, 2). PCs are terminally differentiated immune effectors that develop from B lymphocytes following infection and vaccination, with the highly specialized function to manufacture and secrete antibodies, the effector molecules of humoral immunity. Circulating antibodies can persist in the absence of antigen and provide protection for a lifetime, affording serological memory. Due to their rapid disappearance in the circulation, PCs have long been thought to be short-lived and to maintain serum antibodies by continuous differentiation from B cells. It has recently become clear that PCs may survive and secrete protective antibodies for decades after antigen encounter (3–6).

The nature of the stimulus that triggers PC generation from short-lived antibody secreting cells (ASC) and the molecular programs underlying such transformation are elusive. Long-lived PCs (LLPCs) reside in dedicated niches, mainly located in the bone marrow (BM). Different cell types and signals combine to shape PC survival niches (5, 7). In humans, the phenotype of LLPCs is currently defined as CD19^neg^CD138^+^CD38^hi^, but phenotypic, biological and functional heterogeneity is likely to exist within this population (8). Far from being quiescent, LLPCs display unrivaled immunoglobulin (Ig) secretion that requires unique organelle organization and adaptive proteostatic and metabolic features that excite great curiosity and represent formidable research challenges (9–11). The longevity of PCs, from their generation throughout their maintenance, relies on multiple factors, whose identification and mechanistic details are critical to advance our understanding of adaptive immunity, as well as the pathophysiology of PC-mediated autoimmune and neoplastic disorders (12). This collection highlights fundamental mechanisms of PC longevity in health and disease, namely, multiple myeloma and allergy, as well as novel PC functions and immunophenotypes in nonhuman primates.

Five review articles within this series discuss the extrinsic and intrinsic determinants of PC maintenance with specific focus and from different perspectives, offering a comprehensive and integrated view of LLPCs. Slifka and Amanna discuss the mechanisms underlying how the structural biology of multivalent antigens can induce durable protective immunity by LLPCs compared with monovalent antigens, an issue whose relevance for vaccine design has become tangible with the SARS-CoV-2 pandemic. Lindquist et al. review the dynamic nature of PC niches, the current knowledge on their molecular and cellular composition, and how its changes may influence PC function, with a specific focus on metabolism and new technologies to gauge it over time *in vivo*. Lightman et al. analyze the extrinsic and intrinsic factors of PC longevity within their niche, including continuous niche-generated signals unique to LLPC survival, metabolic fitness, specific bioenergetic cues, and cellular components of the LLPC niche itself. Khodadadi et al. depict a historical perspective on the recent discovery and characterization of PCs. In view of the incessant capacity of LLPCs to secrete antibodies, independently of antigen presence, T cell help or supply from precursors, they propose to call them *memory* PCs. They too discuss the determinants of PC longevity, namely, extracellular components — cellular compartments and soluble and membrane-bound molecular elements — and intracellular factors, related to differentiation and stress-adaptive pathways, metabolism, autophagy, and survival. Since PCs acquire longevity in inflamed tissues, they also review the inflammatory cellular and molecular mechanisms thought to support PC survival. Finally, Nguyen et al. review the molecular, functional and immunophenotypic features that hallmark the transition of human short-lived ASCs to LLPCs and the known cell-autonomous and nonautonomous factors required, with a specific perspective on the adaptive significance of the changes imparted to early minted ASCs as they mature into late BM LLPCs. Of technological relevance, they propose a minimal set of extrinsic conditions, combining secreted factors from BM stromal cells, APRIL and low oxygen tension as an experimental human BM mimic able to maintain human ASCs in culture for weeks for molecular *ex vivo* studies.

Not all PCs are protective. Aberrant PC generation and maintenance can result in pathogenic PCs in human disease, like PC dyscrasias, where transformed PCs gain enhanced proliferation and survival. The prototypical PC cancer is multiple myeloma, an age-onset malignancy characterized by the clonal expansion of PCs at multiple foci in the BM, typically resulting in lytic bone lesions, hypercalcemia, renal failure, anemia, and infections (13). Myeloma cells are the malignant counterpart of BM resident LLPCs; however, the exact cell of origin of this cancer remains unknown. Myeloma cells usurp the BM niche-specified pro-survival signals intended for LLPCs. Targeting such multi-cellular environmental niches holds great therapeutic potential against myeloma, but a comprehensive and translatable knowledge of the underlying circuits warrants more investigation (14). Barwick et al. discuss the cellular and genetic origin of multiple myeloma, reconstructing the milestone discoveries on clonal gammopathies and their interconnectedness with the advancing knowledge of PCs, with in-depth focus of the myeloma-driving genetic and epigenetic alterations in the context of PC differentiation and biology.

IgE PCs arise for protection against parasites, but can mediate allergic diseases. Ramadani et al. deployed a human *ex vivo* tonsil B cell culture system to investigate transcriptional profiles of IgE-expressing PCs and identified putative specific gene expression trajectories and regulatory networks.

Two additional original articles complete this series. PCs have functions beyond Ab secretion (15). Using mouse models, Meng et al. gauged the contribution of PCs to IL-10 provision in the BM. Besides confirming PCs as the chief source of IL-10, they tested its function and demonstrated a key role of IL-10 in driving myeloid lineage differentiation, an effect that appeared to increase with age. Lastly, Zhang et al. defined the surface immunophenotypic markers that identify antibody-secreting plasmablasts in the nonhuman primates, Chinese rhesus macaques. Of biotechnological and therapeutic relevance, this work may help isolate ASCs for efficient mAb cloning and evaluate antibody responses to vaccination or infection in these human-relevant animal species.

In summary, we present a unique collection of review and original articles dissecting the known mechanisms of PCs in health and disease to raise essential questions that still remain at large in the generation and maintenance of LLPCs.

## Author Contributions

All authors have made a substantial, direct and intellectual contribution to the work, and approved it for publication.

## Funding

The authors’ work is supported, in part, by grants from the NIH/NIAID (R01AI121252; P01AI125180; U19AI1109962; U01AI1993) and Gates Innovations Technology (INV-002351) to FE-HL, from NIH/NIAID (R01AI137127; U01AI144673; 5U19AI057266) to JW, and from Fondazione AIRC (Investigator Grant 23245) and Fondazione Cariplo (2018–0541) to SC.

## Conflict of Interest

FE-HL is founder of MicroB-plex, Inc., inventor of patents for plasma cell survival media. FE-HL also acknowledges research funding received from Genentech and the Bill and Melinda Gates Foundation and is a consultant for Pulsar.

The remaining authors declare that the research was conducted in the absence of any commercial or financial relationships that could be construed as a potential conflict of interest.
